# The American Exhibit to Be Made at the Paris World’s Fair

**Published:** 1888-11

**Authors:** 


THE AMERICAN EXHIBIT TO BE MADE AT THE PARIS WORLD'S FAIR NEXT YEAR.—The Universal Exposition of 1889, at Paris, promises to be one of the largest and most successful of the world's fairs held in recent years. Elaborate arrangements for the reception and display of the exhibits are well under way, and unusual facilities both for the transportation of goods from this country and their care are assured. The French government extended a formal invitation to the United States to take part in the exposition. The invitation was accepted by a joint resolution of the Senate and House of Representatives, and the Governors of the several States and Territories were requested to invite the people to assist in the proper representation of the products of American industry and of the natural resources of the country. The President was directed to appoint a commissioner-general and an assistant commissioner-general, to make all the arrangements for exhibit and represent the government at the exposition. He was also directed to appoint nine scientific experts as assistants to the commission, each to be assigned to one of the nine groups into which the exhibits will be divided. Provision was made for the salaries of the commissioners and the necessary assistants, and the sum of $250,000 was appropriated to be used under the direction of the Secretary of State to defray all expenses. The action of Congress was approved May 10, and the President has appointed General William B. Franklin, Commissioner-General, and Mr. Somerville P. Tuck, Assistant Commissioner General.

